# Quantifying Additional Procedures in Functionally Single-Ventricle Disease: A National Cohort Study

**DOI:** 10.1016/j.atssr.2023.12.001

**Published:** 2023-12-20

**Authors:** Qi Huang, Deborah Ridout, Victor Tsang, Nigel E. Drury, Timothy J. Jones, Hannah Bellsham-Revell, Elena Hadjicosta, Anna N. Seale, Chetan Mehta, Christina Pagel, Sonya Crowe, Ferran Espuny-Pujol, Rodney C.G. Franklin, Kate L. Brown

**Affiliations:** 1 Clinical Operational Research Unit, Department of Mathematics, University College London, London, United Kingdom; 2 Population, Policy and Practice Programme, Great Ormond Street Institute of Child Health, University College London, London, United Kingdom; 3 Great Ormond Street Hospital Biomedical Research Centre and Institute of Cardiovascular Science, University College London, London, United Kingdom; 4 Department of Paediatric Cardiology and Cardiac Surgery, Birmingham Children’s Hospital, Birmingham, United Kingdom; 5 Institute of Cardiovascular Sciences, University of Birmingham, Birmingham, United Kingdom; 6 Department of Paediatric Cardiology, Evelina London Children’s Hospital, London, United Kingdom; 7 Department of Paediatric Cardiology, Royal Brompton and Harefield NHS Foundation Trust, London, United Kingdom

## Abstract

**Background:**

Given their importance as a metric for health care evaluation, this study’s aim was to evaluate the rates of surgical and catheter reinterventions for children with functionally single-ventricle (f-SV) congenital heart disease (CHD) undergoing staged palliation.

**Methods:**

We undertook a retrospective cohort study of children born with f-SV CHD between 2000 and 2018 in England and Wales, using the national registry, with survival ascertained in 2020. Competing risk analysis was used to describe the incidence of additional procedures that occurred first, during follow-up, accounting for competing events of death or transplantation.

**Results:**

Of 56,039 patients who received an intervention for CHD, 3307 (5.9%) had f-SV. The largest diagnostic subcategories were hypoplastic left heart syndrome (1266 [38.3%]), tricuspid atresia (448 [13.5%]), and double-inlet left ventricle (328 [9.9%]). During a median follow-up of 5.4 (interquartile range, 0.8-10.8) years, 921 (27.9%) patients had at least 1 additional interstage surgery and 1293 (39.1%) had at least 1 additional interstage catheter intervention. The cumulative incidence of additional surgery at 6 months after stage 1 was 17.6% (95% CI, 16.2%-19.0%); at 2 years after stage 2, 8.3% (7.2%-9.5%); and at 5 years after stage 3, 8.4% (7.0%-9.9%). The cumulative incidence of additional catheter at 6 months after stage 1 was 18.0% (16.6%-19.4%); at 2 years after stage 2, 14.7% (13.3%-16.2%); and at 5 years after stage 3, 23.7% (21.5%-26.0%).

**Conclusions:**

It is important to quantify additional procedures for children with f-SV disease to inform parents and health professionals, potentially facilitating the development of interventions that aim to reduce these important adverse outcomes.

**In Short**

▪ In a population-based study of 3307 children treated for functionally single-ventricle disease in England, the cumulative incidence of additional surgery at 6 months after stage 1 was 17.6% (16.2%-19.0%); at 2 years after stage 2, 8.3% (7.2%-9.5%); and at 5 years after stage 3, 8.4% (7.0%-9.9%).
▪ In addition, the cumulative incidence of additional transcatheter interventions at 6 months after stage 1 was 18.0% (16.6%-19.4%); at 2 years after stage 2, 14.7% (13.3%-16.2%); and at 5 years after stage 3, 23.7% (21.5%-26.0%).

Given anatomic complexity and the need for a series of surgeries to palliate the circulation in functionally single-ventricle (f-SV) congenital heart disease (CHD), additional interventions are frequently needed and have a range of impacts^1^^,^^2^ for these patients. Therefore, we aimed to ascertain and to quantify additional interventions over and above the expected staged palliative pathway of procedures that were undertaken in the whole f-SV population for England and Wales between 2000 and 2018. We questioned what types of additional or off-pathway interventions occurred for patients with f-SV in terms of category and most common types, by diagnostic subgroup and between the 3 surgical stages.

## Patients and Methods

### Study Design

We undertook a retrospective population-based cohort study of all children born in England and Wales with f-SV CHD between 2000 and 2018 who underwent staged palliation using the National Congenital Heart Disease Audit, with survival status from the Office of National Statistics. The study was approved by the UK National Health Service Stanmore Research Ethics Committee (Reference 18/LO/1688), and the need for patient consent was waived.

### Functionally Single-Ventricle Subtypes

We identified patients with f-SV CHD in the cohort as detailed in the Supplemental Figure. Patients were assigned to f-SV diagnosis subgroups (Supplemental Table 1) for which there were at least 100 patients, combining rarer conditions into the “other f-SV” group. Using a hierarchical approach, these were classic hypoplastic left heart syndrome (HLHS),^3^ f-SV with atrial isomerism, double-inlet ventricle, tricuspid atresia, mitral atresia without HLHS, unbalanced common atrioventricular septal defect (AVSD),^4^^,^^5^ pulmonary atresia without other complex features but with f-SV, and other f-SV.

### Outcomes

The primary outcomes were any additional cardiac operation or interventional catheter procedure over and above the treatment pathway, after interventional treatment was started.

### Procedure Types

To identify the primary outcome, we first identified cardiac procedures on the planned treatment pathway (not the study outcome; see Supplemental Table 2). These were pre-pathway procedures after the child’s birth (fetal procedures were not in the data set) and before the first staged surgery: palliative first-stage procedures (stage 1), including surgical, hybrid, and catheter types; bidirectional superior cavopulmonary (Glenn) anastomosis (stage 2) surgery; a combination of aortopulmonary amalgamation and augmentation with construction of a bidirectional superior cavopulmonary anastomosis (comprehensive stage 2); and total cavopulmonary connection procedures or Fontan (stage 3). We note that >1 palliative first-stage procedure may occur in certain patients; but for the purpose of our study analysis, we allowed only a single stage 1 procedure to be counted on the treatment pathway.

### Additional Procedures (Primary Outcome)

We defined and identified additional procedures that contributed to the primary outcome as any that were not identified as before or on the treatment pathway. Additional procedures were all cardiac interventions, inclusive of additional staged procedures required for clinical reasons and reinterventions for residual or recurrent lesions, noting that on the basis of our data sources we could not ascertain indications. We further categorized the additional procedures into surgery (inclusive of operations and procedures involving both surgery and catheters, ie, hybrid types) and catheters (inclusive of interventional catheterizations and procedures involving electrophysiology interventions by catheterization) as detailed in Supplemental Table 3.

### Statistical Methods

We explored the frequency and timing of additional procedures, by intervention type, across the period of follow-up. We identified separate time periods between the first stage 1 procedure and the next staged treatment for patients who completed stage 1; between stage 2 and stage 3 for patients who completed stage 2; and after stage 3 for patients who completed stage 3. Cumulative incidence function was used to describe the incidence of an additional procedure that occurred first during the follow-up period, while accounting for the occurrence of competing events (ie, death and completion of next staged treatment or heart transplant without additional intervention).

The Office of National Statistics data were received 30 months after the most recent procedure reported in the National Congenital Heart Disease Audit data (because of the time required for regulatory processes), so procedures undertaken during this time gap may have been missed. We therefore used patients’ status as of March 31, 2018, to ascertain additional procedures and their competing events.

All statistical analyses were performed with Stata 15 software (StataCorp LLC).

## Results

### Study Population

From the registry population of 56,039 patients, 3307 (5.9%) with f-SV met our inclusion criteria. The cohort consisted of HLHS (1266 [38.3%]), f-SV with atrial isomerism (243 [7.4%]), double-inlet ventricle (328 [9.9%]), tricuspid atresia (448 [13.6%]), mitral atresia without HLHS (112 [3.4%]), unbalanced AVSD (231 [7%]), pulmonary atresia (138 [4.2%]), and others (541 [16.4%]). Patient demographics and clinical variables indicated that 1937 (58.6%) were male, 553 (16.7%) had a congenital noncardiac condition, and 199 (6%) had premature birth (details in Supplemental Table 4).

### Planned Interventions (Not the Study Outcome)

**Figure 1:**
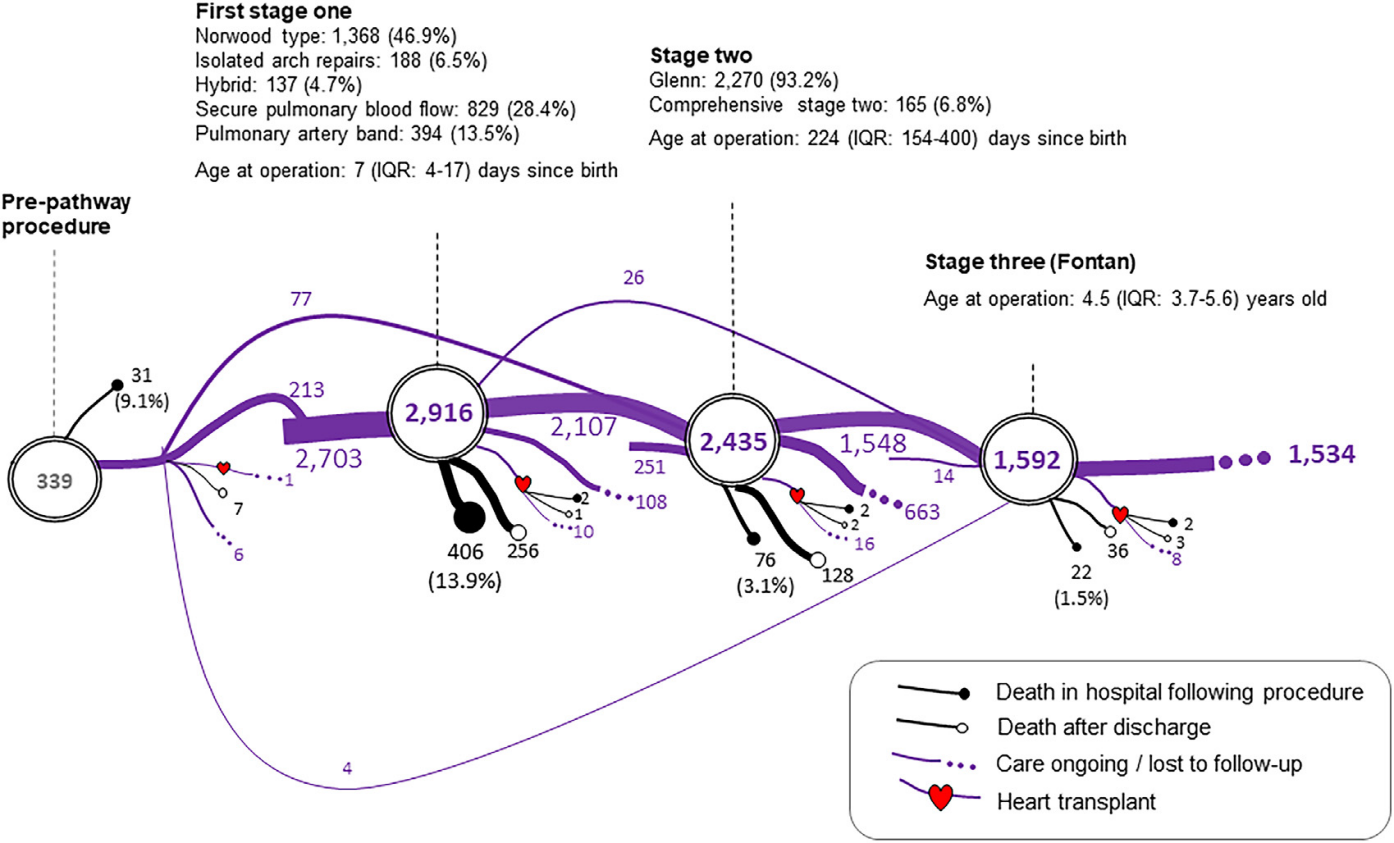
Planned treatment pathways and outcomes (not including additional interventions) for patients born with functionally single ventricle between 2000 and 2018 in England and Wales. The subtypes of staged procedure are presented as number (percentage), where the ratio is computed on the basis of the number of patients who had stage 1, stage 2, or stage 3 procedure. (IQR, interquartile range.)

Figure 1 depicts the serial procedures that were considered to form the treatment pathway for f-SV and captures the number of patients who had undergone each stage when the data set was analyzed. For the 3307 children, treatment pathway included procedures before stage 1 in 339 (10.3%) patients (eg, 195 [5.8%] balloon atrial septostomy; Supplemental Table 5); at least 1 first-stage procedure in 2916 (88.2%) patients (consisting of 1368 [46.9%] Norwood-type operations, 188 [6.5%] isolated arch repairs [with or without pulmonary arterial banding], 137 [4.7%] hybrid procedures for HLHS, 829 [28.4%] procedures to secure pulmonary blood flow [including arterial shunt operations and ductal stenting procedures], and 394 [13.5%] isolated pulmonary arterial banding procedures); a second stage in 2435 (73.6%) patients, 2270 (68.6%) Glenn and 165 (5%) comprehensive stage 2 operations; a third stage (Fontan) in 1592 (48.1%) patients; and heart transplant in 47 (1.4%) patients.

### Additional Procedures (Study Outcome)

**Figure 2:**
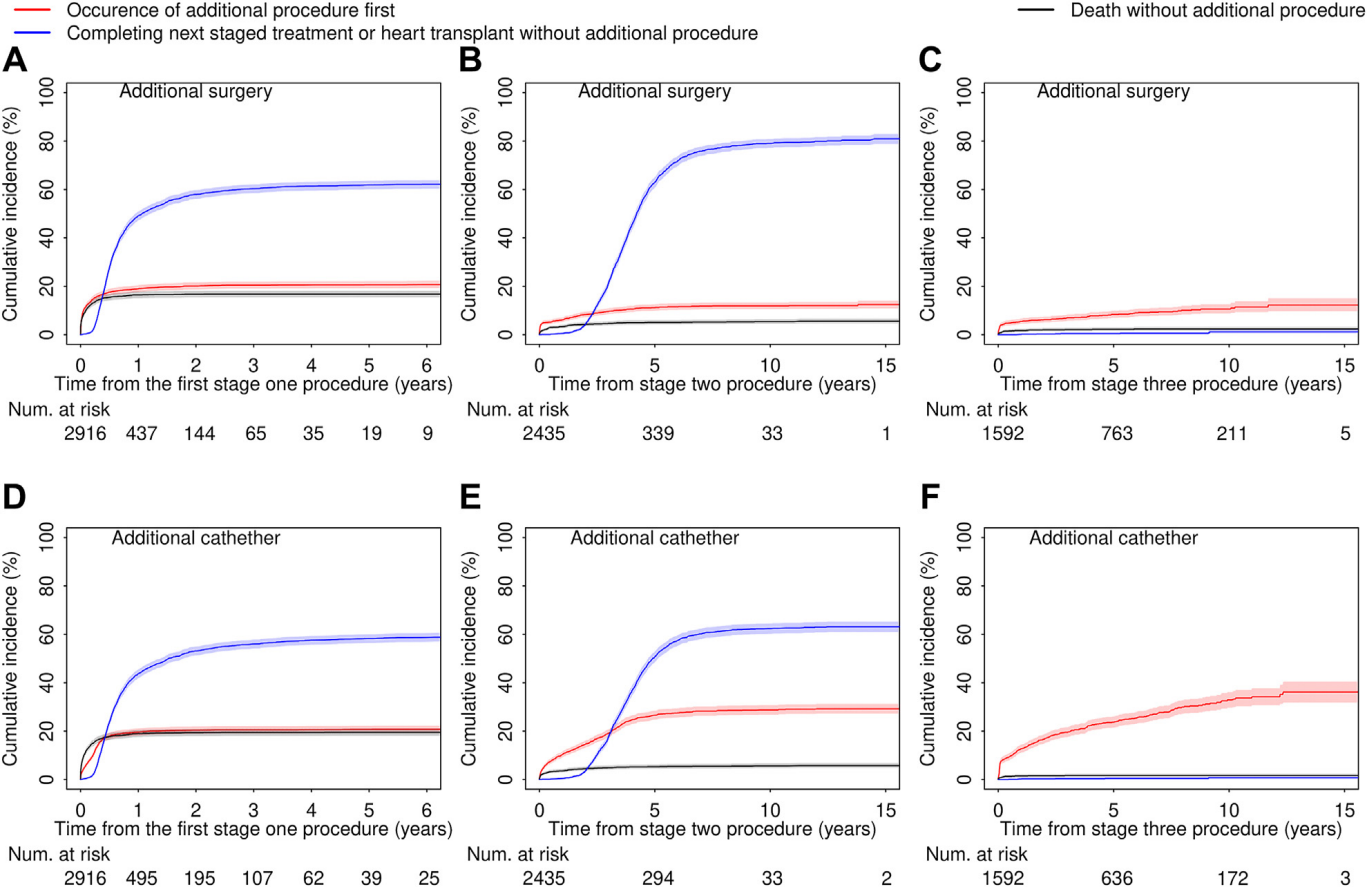
Cumulative incidence with 95% CI of first occurrence of additional procedure and their competing events by surgical stages. (A, D) Cumulative incidence of procedures (surgery or catheters) that occurred between stage 1 and the next staged procedure based on 2916 patients. (B, E) Cumulative incidence of procedures (surgery or catheters) that occurred between stage 2 and stage 3 based on 2435 patients. (C, F) Cumulative incidence of procedures (surgery or catheters) that occurred after stage 3 procedure based on 1592 patients.

During a median follow-up time of 5.4 (interquartile range [IQR], 0.8-10.8) years, 1730 (52.3%) patients had at least 1 additional procedure, of whom 887 (26.8%) had multiple (≥2) additional procedures. We present the frequencies of additional procedures, between the 3 surgical stages by f-SV diagnostic subgroup, in Supplemental Table 6. The proportion of patients who had at least 1 additional procedure was 648 (51.2%) for HLHS, 140 (57.6%) for f-SV with atrial isomerism, 165 (50.3%) for double-inlet ventricle, 224 (50%) for tricuspid atresia, 50 (44.6%) for mitral atresia without HLHS, 114 (49.4%) for unbalanced AVSD, 93 (67.4%) for pulmonary atresia, and 296 (54.7%) for others. We next quantified the frequency and time to occurrence of additional procedures between each stage of treatment (Supplemental Table 7) and the most common procedures (Supplemental Table 8). The proportion of patients having additional surgeries decreased during the planned treatment stages (20.4% after the first stage 1 procedure, 10.9% after stage 2, and 8.5 % after stage 3), whereas the proportions of patients having additional catheters were similar by treatment stage (20.4% after the first stage 1 procedure, 25.3% after stage 2, and 24.3% after stage 3). We illustrate the cumulative incidence of additional procedures as well as their competing events by intervention types and surgical stages in Figure 2.

#### Between First Stage 1 Procedure and Next Staged Treatment

Of 2916 patients who had a stage 1 procedure, 1018 (34.9%) had at least 1 additional procedure before the next stage undertaken, during a median follow-up of 118 days (IQR, 25-230 days). Of these 1501 interstage additional procedures, 774 (51.6%) were surgical in 596 patients and 727 (48.4%) were catheters in 596 patients. The first additional surgery or catheter took place at a median of 20 days (IQR, 3-96 days) and 81 days (27-123 days) after stage 1, respectively. The cumulative incidence of interstage surgery and catheter at 6 months after stage 1 was 17.6% (95% CI, 16.2%-19.0%) and 18.0% (16.6%-19.4%), respectively.

#### Between Stage 2 and Stage 3

Of 2435 patients who had a stage 2 procedure, 751 (30.8%) had at least 1 additional interstage procedure before stage 3, during a median follow-up of 2.8 years (IQR, 1.3-3.9 years). Of these 1198 interstage additional procedures, 350 (29.2%) were surgical in 265 patients and 848 (70.8%) were catheters in 616 patients. The first additional surgery or catheter took place at a median of 205 days (IQR, 8-772 days) and 595 days (118-1154 days) after stage 2, respectively. The cumulative incidence of interstage surgery and catheter at 2 years after stage 2 was 8.3% (95% CI, 7.2%-9.5%) and 14.7% (13.3%-16.2%), respectively.

#### After Stage 3

Of 1592 patients who had a Fontan, 462 (29%) had at least 1 additional procedure after Fontan, during a median follow-up of 3.5 years (IQR, 1.0-7.0 years). Of these 728 additional procedures, 165 (22.7%) were surgery in 135 patients and 563 (77.3%) were catheters in 387 patients. The median duration between stage 3 and the first additional surgery or catheter intervention was 74 days (IQR, 12-987 days) and 345 days (IQR, 26-1118 days), respectively. The cumulative incidence of additional surgery and catheter at 5 years after stage 3 was 8.4% (95% CI, 7.0%-9.9%) and 23.7% (21.5%-26.0%), respectively.

## Comment

In this novel population-based analysis of additional procedures in children born between 2000 and 2018 in England and Wales and treated for f-SV disease, during a median follow-up of 5.4 years, additional procedures were common, affecting 52.3%, with 26.8% of children having multiple additional procedures. Across all stages, 62.4% of additional procedures were interventional catheters; breaking this down, between stages 1 and 2, surgery and catheters made up similar proportions of additional procedures; then, a catheter approach dominated in subsequent stages. We have reported a higher risk of additional cardiac surgery and transcatheter reinterventions between palliative staged surgeries,^6^ based on risk factors of the initial stage 1 approach (eg, hybrid vs Norwood), greater patient complexity in terms of noncardiac comorbidities (eg, genetic syndromes), and additional cardiac problems (eg, impaired ventricular function), and in children who had already had a more complex operative pathway than standard. We conclude that it is important to quantify and to report the frequency of additional procedures for children with f-SV disease to inform parents and health professionals, potentially facilitating the development of interventions that aim to reduce these important adverse outcomes.
