# Supplementary material for: Quantifying Additional Procedures in Functionally Single-Ventricle Disease: A National Cohort Study
Source: Ann Thorac Surg Short Rep. 2023 Dec 20;2(2):282–6. doi: 10.1016/j.atssr.2023.12.001 (PMC11708582; doi:10.1016/j.atssr.2023.12.001)
Supplement: Supplementary Tables [file mmc1.docx]

# Supplementary Materials

## Table S1 Definition of functionally single ventricle (f-SV) congenital heart disease (CHD) patients.

| **f-SV Disease Types** | **Definition of CHD Type** |
| --- | --- |
| **Classic hypoplastic left heart syndrome (HLHS)** | CHD with a small left ventricle, left sided valvar stenosis or atresia, normally related great arteries and no common atrioventricular junction based on diagnostic and procedure codes as reported previously. ^1 2^ |
| **Hearts with an univentricular atrioventricular connection** | 1. Double inlet atrioventricular connection: both double inlet left ventricle (DILV) and double inlet right ventricle (DIRV), whether via two atrioventricular valves or a common atrioventricular valve; 2. Absence of one atrioventricular connection: non HLHS mitral atresia and tricuspid atresia; ^3 4^ |
| **Unbalanced atrioventricular septal defect** | A common atrioventricular junction and valve with only one completely well-developed ventricle (AVSD), but 75% or less commitment to the dominant ventricle, and a f-SV management pathway. |
| **Atrial isomerism with f-SV** | Atrial isomerism with only one well developed ventricle and a f-SV management pathway, irrespective of type of atrioventricular connection. |
| **Other major primary congenital heart diagnoses with f-SV circulation** | CHDs where due to the presence of a hypoplastic ventricle or a straddling atrioventricular valve. The management pathway entailed staged stage one procedures for f-SV.  ^5^ |

## Table S2: Procedures on the established pathway of palliation for functionally single ventricle (f-SV) disease.

BCPA: bidirectional cavopulmonary anastomosis; f-SV=functionally single ventricle; FUH=functionally univentricular hearts; HLHS=hypoplastic left heart syndrome; TCPC: Total cavopulmonary connection.

| **Pre-pathway** |
| --- |
| Procedures that occurred after the child’s birth (foetal procedures were not included) and before the first stage of palliative surgery.^2 4^ |
| **Palliative first stage procedures** |
| Procedures that are routinely the first surgical palliation including:^4 6 7^  Type A) Norwood type operations inclusive of Norwood with Modified R Blalock interposition shunt and with right ventricle to pulmonary artery valveless conduit (Sano) and other Damus-Kaye-Stansel type procedures.  Type B) All types of coarctation and interrupted arch repairs, with or without pulmonary arterial banding.  Type C) HLHS hybrid procedures, involving bilateral pulmonary arterial bands and ductal stenting either at the same procedure or separated by up to 2 weeks as defined by NCHDA.  Type D) procedures to secure pulmonary blood flow, including all types of systemic-to-pulmonary arterial shunts, stent placement in arterial duct (PDA), pulmonary valvotomy and other operations to relieve right ventricular outflow tract obstruction and cardiac catheterisations to perforate or widen the pulmonary valve.  Type E) procedures to protect the pulmonary vascular bed from excessive flow, (e.g.: pulmonary trunk or arterial bands). |
| **Stage two procedures** |
| Glenn - Construction of a bidirectional superior cavopulmonary (Glenn) anastomosis (BCPA), including when BCPA procedures were contemporaneous with other procedures that are sometimes required at the same time (e.g. pulmonary arterial reconstruction).  Comprehensive stage two which is a combination of aorto-pulmonary amalgamation and augmentation with construction of a BCPA. ^4 6 7^ |
| **Stage three procedure** |
| Total cavopulmonary connection procedures (TCPC or Fontan). This included Fontan-type procedures when they occurred contemporaneously with other procedures that are sometimes clinically required at the same time (e.g.: atrioventricular valve repair). ^4 6 7^ |

## Table S3 Assignment of additional off pathway procedures into groups

Non-contributory procedures were removed from the analysis, i.e., diagnostic catheters, extracorporeal life support procedures.

Additional procedures were grouped based on an algorithm first introduced/applied in the annual report of The National Institute for Cardiovascular Outcomes Research (NICOR): “National congenital heart disease audit 2014-17 summary report”.^8^

| **Output of activity algorithm - detailed group** | **Procedure Grouping** |
| --- | --- |
| Bypass cardiac procedures | Surgical and hybrid procedure (Surgery) |
| Non-bypass cardiac procedures | Surgical and hybrid procedure (Surgery) |
| Hybrid involving surgery and catheter procedures | Surgical and hybrid procedure (Surgery) |
| Implantable cardioverter defibrillator (non-surgical) | Procedural catheters and Electrophysiology (Catheter) |
| Pacemaker (non-surgical) | Procedural catheters and Electrophysiology (Catheter) |
| Electrophysiological study (non-surgical) | Procedural catheters and Electrophysiology (Catheter) |

## Table S4 Characteristics of patients with functionally univentricular hearts (f-SV).

Data are n (%) or median value (IQR) based on n=3,307 f-SV patients. Low weight includes imputed data.

| **Patient factor** | |
| --- | --- |
| **Non-cardiac variables** |  |
| Recent data - Born after April 2009 | 1,697 (51.3) |
| Sex: Male | 1,937 (58.6) |
| Prematurity | 199 (6) |
| Congenital non-cardiac comorbidity | 553 (16.7) |
| Low weight baby (below 2.5Kg) at first procedure | 339 (10.3) |
| Acquired comorbidity at first procedure | 159 (4.8) |
| Increased severity of illness at first procedure | 384 (11.6) |
| Age (days) at first procedure | 6 (4-27) |
| **f-SV subtype** |  |
| Hypoplastic left heart syndrome (HLHS) | 1,266 (38.3) |
| f-SV with atrial isomerism | 243 (7.3) |
| Double inlet ventricle | 328 (9.9) |
| Tricuspid atresia | 448 (13.5) |
| Mitral atresia without HLHS | 112 (3.4) |
| Unbalanced atrioventricular septal defect (AVSD) | 231 (7) |
| Pulmonary Atresia without other complex features but with f-SV | 138 (4.2) |
| Other f-SV types | 541 (16.4) |
| **Stage one and subtypes (at first stage one)** |  |
| No stage one procedure | 391 (11.8) |
| Norwood type | 1,368 (41.4) |
| Isolated arch repairs | 188 (5.7) |
| Hybrid procedure with bilateral banding and ductal stent | 137 (4.1) |
| Secure pulmonary blood flow (arterial shunt or right ventricular outflow tract procedure) | 829 (25.1) |
| Pulmonary arterial band | 394 (11.9) |
| Stage one with concurrent surgery | 157 (4.7) |
| **Stage two and subtypes** |  |
| No stage two procedure | 872 (26.4) |
| Glenn | 2,270 (68.6) |
| Comprehensive stage two | 165 (5) |
| Stage two with concurrent surgery | 951 (28.7) |
| **Stage three** |  |
| No stage three procedure | 1,445 (51.9) |
| Stage three (Fontan) | 1,592 (48.1) |
| Stage three with concurrent surgery | 257 (7.8) |
| **Additional cardiac risk factor (any time point)** | 234 (7.1) |

## Table S5: Frequency of pre-pathway procedures.

The top 5 most common pre-pathway procedures (procedures that occurred before the first staged treatment) in f-SV patients. Procedures may arise more than once in the same patient. Data are n(%) based on n=3,307 f-SV patients.

| Surgery | Atrial septectomy. | 37 (8.4) |
| --- | --- | --- |
|  | Application of right & left pulmonary arterial bands. | 27 (6.1) |
|  | Patent arterial duct (PDA) closure: surgical. | 21 (4.8) |
|  | Totally anomalous pulmonary venous connection repair. | 18 (4.1) |
|  | Pacemaker system placement: permanent epicardial. | 6 (1.4) |
| Catheters | Balloon atrial septostomy by pull back (Rashkind). | 205 (46.4) |
|  | Interatrial communication creation-enlargement. | 24 (5.4) |
|  | Balloon dilation of aortic valve. | 22 (5) |
|  | Transluminal radiofrequency ablation procedure for arrhythmia. | 4 (0.9) |
|  | Blade atrial septostomy. | 4 (0.9) |

## Table S6: Number of additional procedures or off-pathway by surgical stages.

| Data are n (%) | | Hypoplastic left heart syndrome (HLHS) | f-SV with atrial isomerism | Double inlet ventricle | Tricuspid atresia | Mitral atresia without HLHS | Unbalanced atrioventricular septal defect | Pulmonary Atresia | Other f-SV | All f-SV cohort |
| --- | --- | --- | --- | --- | --- | --- | --- | --- | --- | --- |
| The whole follow-up. n=3,307 patients. | | | | | | | | | | |
| Number of Patients | | 1,266 | 243 | 328 | 448 | 112 | 231 | 138 | 541 | 3,307 |
| Number of additional procedures | None | 618 (48.8) | 103 (42.4) | 163 (49.7) | 224 (50) | 62 (55.4) | 117 (50.6) | 45 (32.6) | 245 (45.3) | 1577 (47.7) |
|  | 1 | 291 (23.0) | 67 (27.6) | 83 (25.3) | 127 (28.3) | 25 (22.3) | 62 (26.8) | 33 (23.9) | 155 (28.7) | 843 (25.5) |
|  | ≥2 | 357 (28.2) | 73 (30.0) | 82 (25.0) | 97 (21.7) | 25 (22.3) | 52 (22.5) | 60 (43.5) | 141 (26.1) | 887 (26.8) |
| Between the first stage one and next staged treatment (based on n=2,916 patients who had a stage one procedure) | | | | | | | | | | |
| Number of Patients | | 1,240 | 190 | 260 | 359 | 94 | 205 | 134 | 434 | 2,916 |
| Number of additional procedures | None | 819 (66.0) | 122 (64.2) | 185 (71.2) | 238 (66.3) | 63 (67.0) | 141 (68.8) | 63 (47.0) | 267 (61.5) | 1,898 (65.1) |
|  | 1 | 292 (23.5) | 46 (24.2) | 48 (18.5) | 82 (22.8) | 24 (25.5) | 50 (24.4) | 40 (29.9) | 121 (27.9) | 703 (24.1) |
|  | ≥2 | 129 (10.4) | 22 (11.6) | 27 (10.4) | 39 (10.9) | 7 (7.4) | 14 (6.8) | 31 (23.1) | 46 (10.6) | 315 (10.8) |
| Between stage two and stage three (based on n=2,435 patients who had a stage two procedure) | | | | | | | | | | |
| Number of Patients | | 776 | 184 | 287 | 358 | 87 | 141 | 128 | 474 | 2,435 |
| Number of additional procedures | None | 502 (64.7) | 122 (66.3) | 209 (72.8) | 258 (72.1) | 68 (78.2) | 97 (68.8) | 91 (71.1) | 337 (71.1) | 1,684 (69.2) |
|  | 1 | 163 (21.0) | 29 (15.8) | 48 (16.7) | 71 (19.8) | 12 (13.8) | 25 (17.7) | 27 (21.1) | 93 (19.6) | 468 (19.2) |
|  | ≥2 | 111 (14.3) | 33 (17.9) | 30 (10.5) | 29 (8.1) | 7 (8.0) | 19 (13.5) | 10 (7.8) | 44 (9.3) | 283 (11.6) |
| Post stage three duration (based on n=1,592 patients who had a stage three procedure) | | | | | | | | | | |
| Number of Patients | | 477 | 106 | 204 | 261 | 62 | 82 | 82 | 318 | 1,592 |
| Number of additional procedures | None | 306 (64.2) | 70 (66.0) | 147 (72.1) | 211 (80.8) | 42 (67.7) | 53 (64.6) | 59 (72.0) | 242 (76.1) | 1,130 (71.0) |
|  | 1 | 98 (20.5) | 24 (22.6) | 41 (20.1) | 29 (11.1) | 13 (21) | 16 (19.5) | 12 (14.6) | 56 (17.6) | 289 (18.2) |
|  | ≥2 | 73 (15.3) | 12 (11.3) | 16 (7.8) | 21 (8) | 7 (11.3) | 13 (15.9) | 11 (13.4) | 20 (6.3) | 173 (10.9) |

## Table S7: Frequency and time of the occurrence of additional procedures and their competing events by surgical stages

Time of event: the days between the occurrence of event and staged procedure beforehand.

Additional procedure: additional procedure occurred first; Death: death without additional procedure; Next stage/Heart transplant: proceed to next staged treatment or heart transplant without additional procedure; Censoring: loss to follow-up without additional procedure. Status of additional procedure and their competing events were computed based on their status as of March 31 2018 (the date when NCHDA data were harvested).

IQR= interquartile range.

| Events | | Frequency (%) | Time of event: median days (IQR) |
| --- | --- | --- | --- |
| Between the first stage one and next staged treatment (based on n=2,916 patients who had stage one). Median age of first stage one operation: 7 days (IQR 4-17). | | | |
| Additional surgery | Additional procedure | 596 (20.4) | 20 (3-96) |
|  | Death | 483 (16.6) | 18 (1-66) |
|  | Next stage/Heart transplant | 1,756 (60.2) | 196 (138-319) |
|  | Censoring | 81 (2.8) | 84 (20-226) |
| Additional Catheters | Additional procedure | 596 (20.4) | 81 (27-123) |
|  | Death | 563 (19.3) | 19 (2-67) |
|  | Next stage/Heart transplant | 1,667 (57.2) | 211 (142-375) |
|  | Censoring | 90 (3.1) | 96 (19-331) |
| Between stage two and stage three (based on n=2,435 patients who had stage two). Median age of stage two operation: 224 days (IQR 154-400). | | | |
| Additional surgery | Additional procedure | 265 (10.9) | 205 (8-772) |
|  | Death | 122 (5.0) | 131 (19-463) |
|  | Next stage/Heart transplant | 1,450 (59.5) | 1,316 (1,021-1,636) |
|  | Censoring | 598 (24.6) | 881 (405-1,358) |
| Additional Catheters | Additional procedure | 616 (25.3) | 595 (118-1,154) |
|  | Death | 129 (5.3) | 96 (14-466) |
|  | Next stage/Heart transplant | 1,181 (48.5) | 1,303 (1,012-1,632) |
|  | Censoring | 509 (20.9) | 810 (377-1,318) |
| Post stage three duration (based on n=1,592 patients who had stage three). Median age of stage three operation: 4.5 years (IQR 3.7-5.6). | | | |
| Additional surgery | Additional procedure | 135 (8.5) | 74 (12-987) |
|  | Death | 34 (2.1) | 58 (10-246) |
|  | Heart transplant | 9 (0.6) | 902 (368-1,929) |
|  | Censoring | 1,414 (88.8) | 1,943 (979-3,040) |
| Additional Catheters | Additional procedure | 387 (24.3) | 345 (26-1,118) |
|  | Death | 26 (1.6) | 46 (3-96) |
|  | Heart transplant | 7 (0.4) | 368 (315-1,298) |
|  | Censoring | 1,172 (73.6) | 1,802 (910-2,974) |

## Table S8: Most common additional procedures or off-pathway by surgical stages

The top 5 most common additional procedures in f-SV patients by surgical stages. Data are n(%). Procedures may arise more than once in the same patient.

| Additional procedure between the first stage one and next staged treatment (ratio computed based on n=2,916 patients who had stage one procedure). | | |
| --- | --- | --- |
| Additional surgery | Modified right Blalock interposition shunt. | 119 (4.1) |
|  | Atrial septectomy. | 86 (3.0) |
|  | Pulmonary trunk band (PA band). | 80 (2.7) |
|  | Norwood type procedure. | 60 (2.1) |
|  | Procedure involving constructed cardiac conduit-shunt. | 56 (1.9) |
| Additional catheters | Balloon dilation of aortic recoarctation. | 157 (5.4) |
|  | Balloon atrial septostomy by pull back (Rashkind). | 119 (4.1) |
|  | Stent placement in cardiac conduit. | 70 (2.4) |
|  | Balloon dilation of left pulmonary artery. | 54 (1.9) |
|  | Stent placement in arterial duct (PDA). | 41 (1.4) |
| Additional procedure between the stage two and stage three (ratio computed based on n=2,435 patients who had stage two procedure). | | |
| Additional surgery | Pulmonary arterioplasty/ reconstruction: central (proximal to hilar bifurcation). | 30 (1.2) |
|  | Pulmonary arterioplasty/ reconstruction. | 28 (1.2) |
|  | Pacemaker system placement: permanent epicardial. | 28 (1.2) |
|  | Redo of bidirectional superior cavopulmonary (Glenn) anastomosis. | 28 (1.2) |
|  | Takedown of Glenn. | 28 (1.2) |
| Additional catheters | Balloon dilation of aortic recoarctation. | 133 (5.5) |
|  | Balloon dilation of left pulmonary artery. | 93 (3.8) |
|  | Transluminal occlusion of systemic-to-pulmonary collateral artery(ies) (MAPCA(s)) with coil-device. | 90 (3.7) |
|  | Venovenous collateral occlusion with device. | 74 (3.0) |
|  | Balloon dilation of aortic recoarctation. | 56 (2.3) |
| Additional procedure post stage three (ratio computed based on n=1,592 patients who had stage three procedure). | | |
| Additional surgery | Pacemaker system placement: permanent epicardial. | 32 (2.0) |
|  | Pulse generator box replacement. | 20 (1.3) |
|  | Fenestration of Fontan type connection. | 19 (1.2) |
|  | Pacemaker system placement: dual chamber. | 11 (0.7) |
|  | Atrial septectomy, | 10 (0.6) |
| Additional catheters | Right atrial septum-tunnel fenestration closure with transluminal device. | 126 (7.9) |
|  | Stent placement in left pulmonary artery. | 89 (5.6) |
|  | Transluminal fenestration of atrial septum-tunnel. | 60 (3.8) |
|  | Balloon dilation of left pulmonary artery. | 37 (2.3) |
|  | Transluminal occlusion of systemic-to-pulmonary collateral artery(ies) (MAPCA(s)) with coil-device. | 33 (2.0) |

## Figure S1 Inclusions and exclusions.

The process of case ascertainment of the study cohort of 3,307 patients with functionally univentricular hearts (f-SV) disease from the National Congenital Heart Diseases Audit (NCHDA) data set with specific exclusions stated at each step.

AVSD=atrioventricular septal defect; f-SV=functionally single ventricle; HLHS=hypoplastic left heart syndrome.


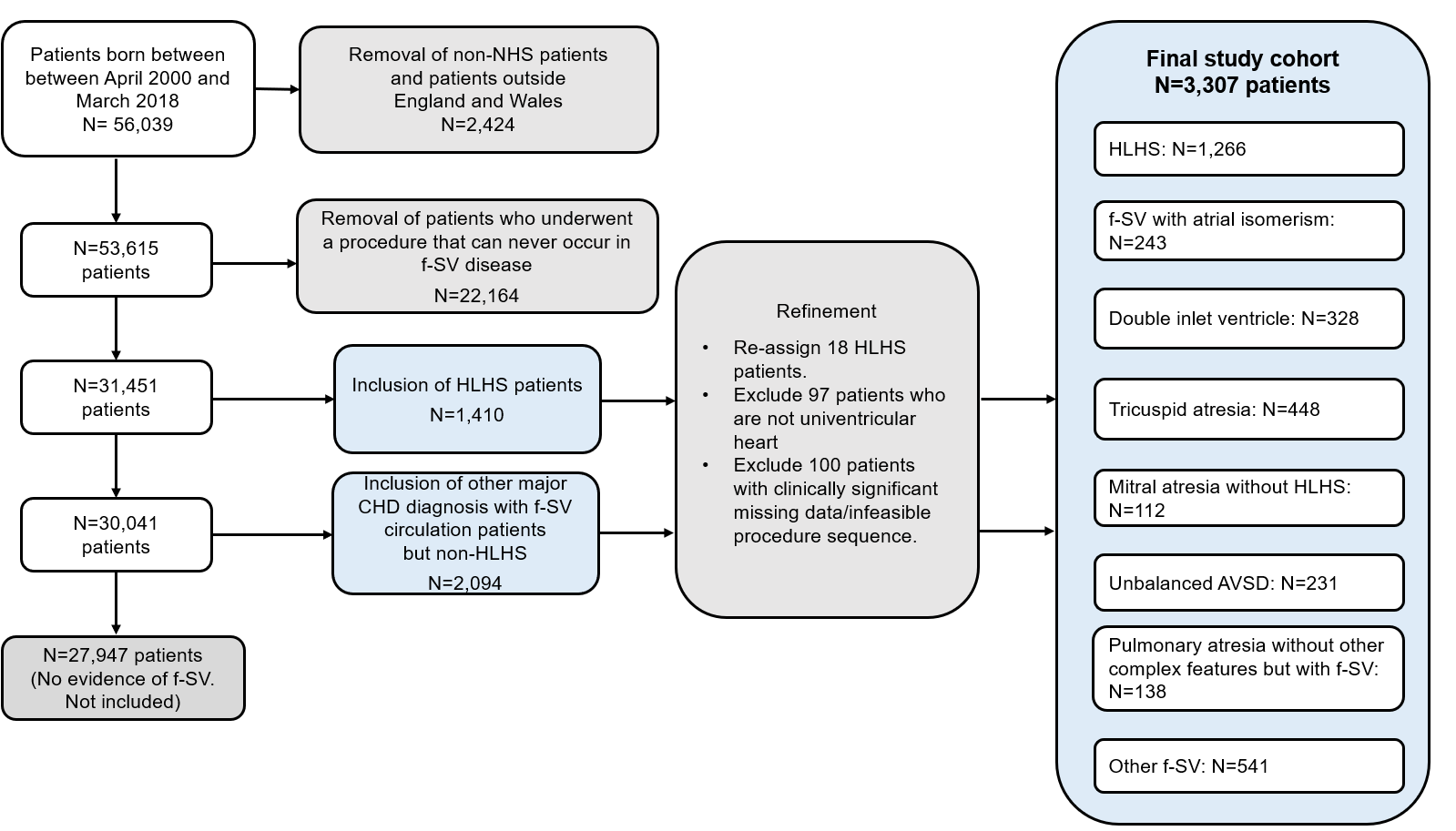


## Reference

1. Rogers, L, Pagel, C, Sullivan, ID, et al. Interventional treatments and risk factors in patients born with hypoplastic left heart syndrome in England and Wales from 2000 to 2015. *Heart*, 2018;104(18): 1500-1507.
2. Rogers L, Pagel C, Sullivan ID, et al. Interventions and Outcomes in Children With Hypoplastic Left Heart Syndrome Born in England and Wales Between 2000 and 2015 Based on the National Congenital Heart Disease Audit. *Circulation* 2017;136(18):1765-67.
3. Jacobs JP, Franklin RC, Jacobs ML, et al. Classification of the functionally univentricular heart: unity from mapped codes. *Cardiol Young* 2006;16 Suppl 1:9-21.
4. Hadjicosta E, Franklin R, Seale A, et al. Cohort study of intervened functionally univentricular heart in England and Wales (2000-2018). *Heart* 2022;108(13):1046-54.
5. Tchervenkov CI, Jacobs ML, Del Duca D. Surgery for the functionally univentricular heart in patients with visceral heterotaxy. *Cardiol Young* 2006;16 Suppl 1:72-9.
6. Feinstein JA, Benson DW, Dubin AM, et al. Hypoplastic left heart syndrome: current considerations and expectations. J Am Coll Cardiol 2012;59(1 Suppl):S1-42.
7. Brown KL, Huang Q, Hadjicosta E, et al. Long-term survival and center volume for functionally single-ventricle congenital heart disease in England and Wales. *J Thorac Cardiovasc Surg* 2022; 166(2):306-316.
8. National congenital heart disease audit 2014-17 summary report: *The National Institute for Cardiovascular Outcomes Research (NICOR)*, 2018.
